# Polo-like kinase inhibition as a therapeutic target in acute myeloid leukemia

**DOI:** 10.18632/oncotarget.27919

**Published:** 2021-06-22

**Authors:** Jan Philipp Bewersdorf, Amer M. Zeidan

**Affiliations:** ^1^Department of Internal Medicine, Section of Hematology, Yale School of Medicine, New Haven, CT, USA; ^2^Cancer Outcomes, Public Policy, and Effectiveness Research (COPPER) Center, Yale University, New Haven, CT, USA

**Keywords:** acute myeloid leukemia, AML, PLK-1 inhibitor, onvansertib, volasertib

## INTRODUCTION

The prognosis of patients with relapsed or refractory (R/R) acute myeloid leukemia (AML) is poor with median overall survival (OS) on the order of months especially in older and unfit patients [1, 2]. While the approval of targeted agents such as FLT3 and IDH inhibitors in R/R AML patients with these mutations has offered new therapeutic options, such druggable mutations are only present in less than half of AML patients [3–6], and even such patients typically progress within months of receiving these therapies highlighting the high unmet clinical need in R/R AML.

**Figure 1:**
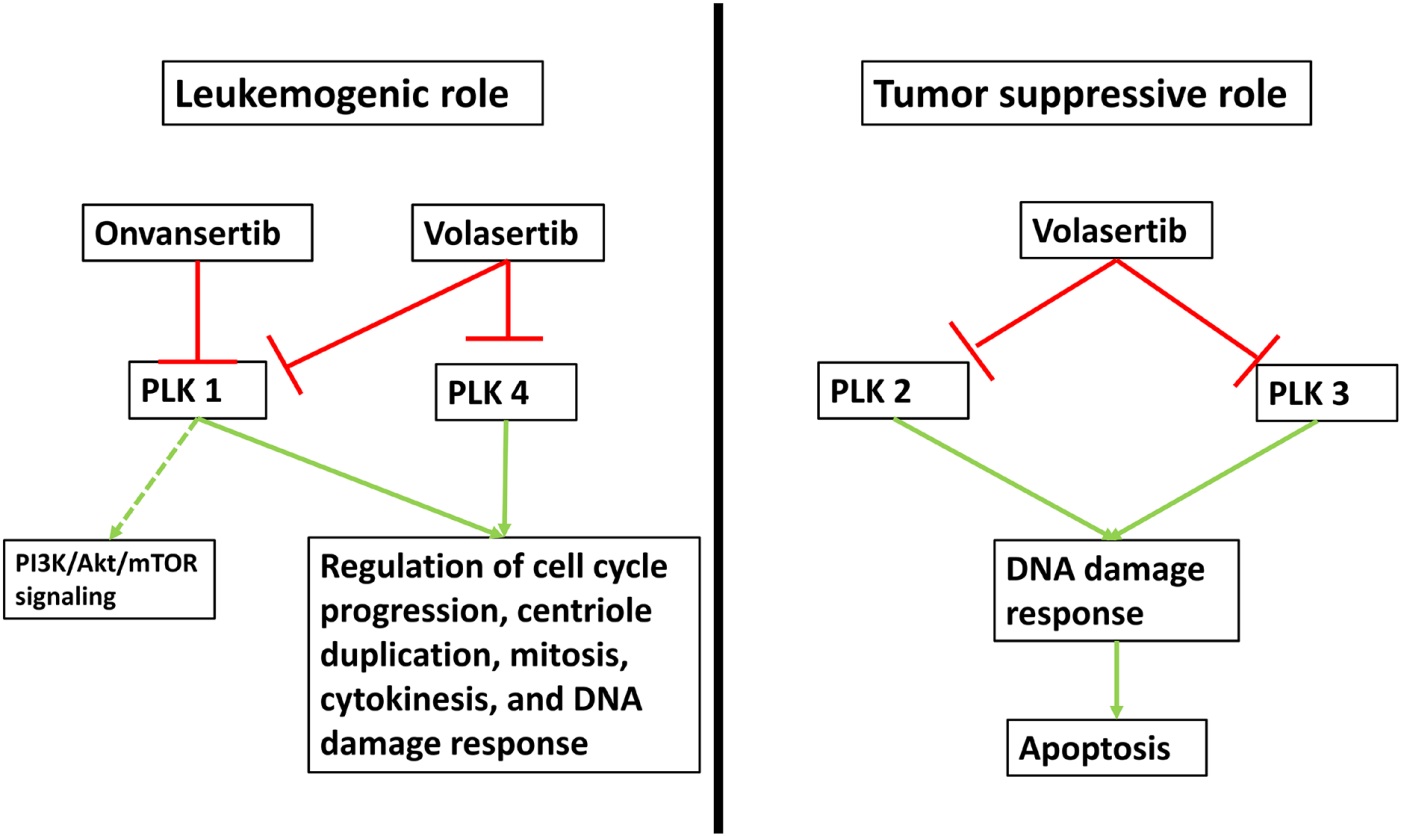
Mechanism of action of PLK inhibition. Five isoforms of polo-like kinases (PLK) have been identified with PLK 1-4 having been associated with a potential role in cell cycle regulation and tumorigenesis. PLK5 is an inactive kinase, almost exclusively expressed in the brain and no studies have associated it with leukemogenesis. PLK1 overexpression has been documented in AML specimens and it functions primarily in the regulation of cell cycle progression, centriole duplication, mitosis, cytokinesis, and DNA damage response. Less is known about its role in the phosphatidylinositol 3-kinase (PI3K)/protein kinase B (Akt)/mammalian target of rapamycin (mTOR) signaling pathway. PLK1 can be inhibited specifically by onvansertib, while volasertib is a pan-PLK inhibitor. Similarly, PLK4 has been linked to mitotic processes and overexpression in solid malignancies has been documented. However, its role in AML is less clear. Conversely, PLK2 and PLK3 expression is increased in response to DNA damage and activation can lead to mitotic arrest and apoptosis. However, preclinical data showing increased methylation and thereby inactivation of PLK2 is associated with a favorable prognosis in AML suggesting a potential pathogenic role of PLK2 in AML.

Polo-like kinases (PLK) play an essential role in the regulation of mitosis and cell cycle processes (Figure 1) [7, 8]. Specifically, PLK1 has been shown to be upregulated in AML and preclinical studies targeting this enzyme have demonstrated induction of cell cycle arrest and apoptosis in cancer cells, especially leukemia cells [7–9]. Among PLK inhibitors, the pan-PLK inhibitor volasertib has been studied most extensively in the clinic. While the addition of volasertib to low-dose cytarabine (LDAC) improved OS in newly-diagnosed unfit AML patients compared to LDAC alone in a randomized phase 2 trial, those results could not be replicated in a larger subsequent phase 3 trial [10]. Onvansertib is an ATP-competitive PLK1-selective inhibitor with a shorter half-life than volasertib which exhibited antitumor activity in both solid and hematologic cancer models including AML xenografts [11, 12]. Additionally, onvansertib showed synergistic activity with cytarabine *in vitro* [11, 12].

Based on these preclinical data, phase 1 clinical trials in patients with R/R AML combining volasertib or onvansertib with the hypomethylating agent (HMA) decitabine or LDAC were conducted [13–15]. Zeidan et al. enrolled a total of 40 patients across 9 centers in the United States with R/R-AML in a phase 1 trial with 17 and 23 patients receiving escalating doses of onvansertib + LDAC or onvansertib + decitabine, respectively. The most common grade 3/4 adverse events were, as expected, hematologic in nature with cytopenias with only 2 (5%) patients requiring dose reductions, and 3 (7.5%) patients treatment discontinuation due to adverse events. With the limitations of a small sample size and absence of a control arm, 33% of patients in the decitabine arm and 13% in the LDAC arm achieved an overall response (defined as complete remission [CR], CR with incomplete hematopoietic recovery [CRi], morphologic leukemia free-state, or partial response) [15]. In correlative studies the authors also showed that changes in circulating tumor DNA (ctDNA) mutant allele frequency (MAF) after the first cycle were predictive of subsequent morphologic responses (CR/CRi) with positive and negative predictive values of 75% (95% CI: 60–100%) and 100% (95% CI: 93–100%), respectively [15]. Additionally, inhibition of phosphorylation of the PLK1 substrate TCTP in circulating blasts while receiving treatment with onvansertib was predictive of blast clearance. Notably, this target engagement was independent of onvansertib dose, pharmacokinetics and the combination partner used [15].

In another phase 1 study, Cortes et al. combined volasertib with decitabine in 13 patients with newly-diagnosed or R/R-AML who were older than 65 years of age and were treated with escalating doses of volasertib and standard-dose decitabine [14]. All patients developed grade 3 or higher adverse events and 2 patients died due to adverse events while on treatment (anemia and acute myocardial infarction) [14]. Three patients (23.1%) achieved an objective response but no patient achieved CR.

The role of PLK inhibitors continues to be explored in myeloid malignancies and more data especially focusing on biomarkers for clinical benefit are needed [14, 16]. It is important to note that PLK inhibitors exhibit important differences and therefore results from one should not be extrapolated to other agents. Compared to volasertib, onvansertib offers the advantage of high specificity for PLK1 and a shorter half-life which might improve the ability to mitigate myelosuppressive toxicities [15]. While venetoclax-based combinations have become the standard of care for newly-diagnosed unfit AML patients, the therapy options in the R/R setting remain very limited [17]. In R/R AML, overall response rates of venetoclax-based combinations with HMA or LDAC were 38.7% in a recent meta-analysis but up to 62% in a phase 2 trial of venetoclax in combination with 10-days of decitabine [18, 19]. However, despite high response rates to venetoclax-based therapies, those therapies are not curative and treatment options for patients progressing on venetoclax are desperately needed, especially in the absence of targetable mutations. Preclinical studies have shown activity of onvansertib in the venetoclax-resistant subcutaneous model OCI-AML3 [20]. Correlative studies from the onvansertib trial also suggested that upregulation of oxidative phosphorylation pathways at baseline was associated with response to onvansertib [21]. As especially leukemic stem cells are dependent on oxidative phosphorylation for their survival, the combination of onvansertib with venetoclax and HMA might have synergistic effects and could be an option to be explored in future clinical trials [22]. Although only 2 patients with CR/CRi in the decitabine + onvansertib arm had been previously treated with decitabine for their preceding myelodysplastic syndrome, this suggests that prior treatment with HMA does not preclude a response suggesting onvansertib can re-sensitize AML cells to HMA therapy. However, it is not clear if the same clinical activity in this setting could have been obtained with onvansertib monotherapy. Several clinical trials exploring PLK inhibitors in AML are currently ongoing (Table 1).

**Table 1:** Overview of active clinical trials of PLK inhibitors in AML

Agent(s)	Phase	Population	NCT identifier
**Onvansertib + LDAC or decitabine**	I/II	R/R AML	NCT03303339
**Volasertib +/– cytarabine**	II	R/R AML or frontline AML ineligible for intensive chemotherapy	NCT00804856
**Volasertib or placebo + LDAC**	III	Newly diagnosed AML ≥ 65 years ineligible for intensive chemotherapy	NCT01721876
**CFI-400945 (oral PLK 4 inhibitor)**	I/II	R/R-AML; MDS and CMML (both HMA failure and newly diagnosed high-risk)	NCT04730258
	I	R/R AML or MDS	NCT03187288
**CYC140 (oral PLK1 inhibitor)**	I	R/R acute leukaemias or MDS	NCT03884829

Abbreviations: AML, acute myeloid leukaemia; CMML, chronic myelomonocytic leukaemia; HMA, hypomethylating agent; LDAC, lowdose cytarabine; MDS, myelodysplastic syndrome; PLK, polo-like kinase; R/R, relapsed/refractory.

Another important finding from the study by Zeidan et al. is the high concordance of MAF in the bone marrow and ctDNA and its association with treatment responses. While this needs to be replicated in larger studies, it suggests the intriguing potential of serial ctDNA assessments to predict clinical responses and potentially guide treatment selection and minimize the need for invasive bone marrow assessments in both clinical trials and routine practice.

Ongoing preclinical research has also suggested synergistic effects of PLK inhibition with the proteasome inhibitor bortezomib and the BET inhibitor BI 894999, which could offer additional options for future clinical trials [23, 24]. Efficacy of onvansertib with both conventional cytotoxic chemotherapy in colorectal cancer and abiraterone-prednisone in castration-resistant prostate cancer has also been demonstrated in early phase clinical trials [25, 26]. In an increasingly individualized and molecularly driven approach to patient care, it will be important to identify biomarkers predicting response to certain therapeutic modalities including PLK inhibitors. Especially patients with complex karyotypes and *TP53* mutations continue to pose a substantial clinical challenge with limited therapeutic options [27]. *In vitro* and xenotransplant models suggested that AML with complex karyotype might be especially vulnerable to PLK1 inhibition providing scientific rationale for dedicated trials in this patient subpopulation [28]. Additionally, post-hoc analyses from the onvansertib + decitabine study showed that 8 of the 10 patients with mutations in the splicing factors *SRSF2* or *SF3B1* had an objective response including four patients with a CR [29]. Beyond AML, PLK1 inhibition might have a role in chronic myelomonocytic leukemia (CMML) with higher expression of *PLK1* in patients with RAS-pathway mutations [30]. Additionally, PLK1 inhibition reduced hepatosplenomegaly and monocytosis with concurrent improvement in hematopoiesis in *RAS*-mutant CMML xenograft models, which could lead to future applications of PLK1 inhibitors in this disease [30]. More clinical and preclinical data remain needed to better define the safety, efficacy, and predictive biomarkers for PLK inhibitors in myeloid malignancies.
